# Early recognition of life-threatening otolaryngologic complications in pediatric epstein-barr virus infectious mononucleosis: a systematic review and meta-analysis

**DOI:** 10.3389/fped.2026.1980308

**Published:** 2026-10-08

**Authors:** Sara Torretta, Giulia De Lucia, Silvia Cavalera, Lorenzo Cavaleri, Ludovica Battilocchi

**Affiliations:** 1 Fondazione IRCCS Ca' Granda Ospedale Maggiore Policlinico, Department of Otorhinolaryngology, Milan, Italy; 2 Department of Clinical Sciences and Community Health, University of Milan, Milan, Italy

**Keywords:** children, EBV, infectious mononucleosis, peritonsillar abscess, upper airway obstruction

## Abstract

**Objective:**

To systematically synthesize evidence on life-threatening otolaryngologic (ENT) complications of pediatric Epstein–Barr virus (EBV) infectious mononucleosis (IM), quantify clinically compatible upper-airway outcomes, assess certainty, and develop a cautious evidence-informed clinical framework.

**Methods:**

PubMed/MEDLINE was searched from January 1, 1990 to September 8, 2026 in accordance with PRISMA 2020. Eligible English-language observational studies and case series included at least four patients and reported severe ENT manifestations of EBV-IM. Evidence was classified as direct pediatric when the analyzed cohort was restricted to patients aged ≤18 years and as mixed-age supportive when relevant pediatric/adolescent observations were embedded within a broader age range and could not be fully separated. Mixed-age studies did not contribute to the primary pediatric meta-analysis. Design-specific JBI tools and outcome-level GRADE were used. Quantitative pooling was restricted to studies with clinically compatible populations, denominators, and outcome definitions.

**Results:**

Nine studies were included. Clinically significant UAO/airway compromise was the only outcome supporting a narrowly defined meta-analysis. Two directly pediatric hospitalized IM cohorts yielded a REML random-effects pooled proportion of 20.8% (95% CI 7.8–44.9%; I^2^ = 69.1%; *τ*^2^ = 0.465; k = 2). Adding one mixed-age cohort in sensitivity analysis yielded 23.2% (95% CI 14.5–35.1%; I^2^ = 38.9%; *τ*^2^ = 0.091; k = 3). Suppurative/deep neck complications, candidate warning features, biomarkers, imaging findings, and treatment effects were not pooled because populations, denominator directions, sampling frames, or outcome definitions were incompatible. Certainty was low that UAO is a clinically important complication in hospitalized pediatric IM and very low for the pooled numerical proportion and the remaining evidence streams.

**Conclusions:**

UAO is the most consistently documented life-threatening ENT manifestation of pediatric EBV-IM, while suppurative/deep neck complications are clinically important but quantitatively heterogeneous. By separating directly pediatric evidence from mixed-age supportive data and preserving outcome-specific denominators, this review provides a structured synthesis of the limited evidence to support clinical awareness and hypothesis generation, while defining priorities for prospective pediatric validation.

## 1 Introduction

Although infectious mononucleosis (IM) is generally regarded as a benign and self-limiting disease, delayed recognition of life-threatening otolaryngologic (ENT) complications remains one of the few potentially preventable causes of severe morbidity in children. While most pediatric patients recover with supportive care alone, a clinically relevant minority experience rapid progression from uncomplicated pharyngotonsillitis to critical upper airway obstruction (UAO), suppurative complications including deep neck infection, or severe immune-mediated complications requiring emergency multidisciplinary management (1, 2). In these patients, early recognition of clinical deterioration may facilitate timely airway protection, appropriate diagnostic work-up, and coordinated multidisciplinary assessment.

ENT manifestations represent the most clinically relevant features of symptomatic IM and account for a substantial proportion of pediatric emergency referrals. Progressive adenotonsillar enlargement may critically reduce upper airway patency, particularly in younger children whose limited anatomical reserve predisposes them to rapid respiratory deterioration. Consequently, progressive dysphagia, drooling, muffled voice, inspiratory stridor, increasing respiratory effort, and inability to maintain adequate oral intake should be regarded as clinically significant warning signs rather than expected manifestations of uncomplicated viral pharyngotonsillitis (3–5).

Suppurative complications, namely deep neck space infections, peritonsillar, parapharyngeal, and retropharyngeal abscesses, represent another major diagnostic challenge. The pathogenesis of these complications is thought to reflect the interplay between Epstein–Barr (EBV)-induced lymphoid hyperplasia, transient immune dysregulation, and secondary bacterial superinfection, which together may facilitate the development of suppurative infections (1, 6). Because uncomplicated IM and suppurative bacterial complications frequently share overlapping clinical, laboratory, and radiological features, early diagnosis may be challenging and is often delayed until cross-sectional imaging or surgical intervention becomes necessary (6–8). Such diagnostic uncertainty may delay appropriate airway protection, timely imaging, antimicrobial therapy, and definitive surgical management.

EBV infection may also trigger hemophagocytic lymphohistiocytosis (HLH), an uncommon but life-threatening systemic hyperinflammatory syndrome. Although HLH is not an ENT complication, disproportionate systemic deterioration in a child initially managed for severe EBV-IM may require its prompt recognition and multidisciplinary assessment (9, 10).

Despite the clinical importance of these complications, the available evidence remains fragmented and methodologically heterogeneous. Most of the current literature derives from retrospective single-center cohorts or case series characterized by relatively small sample sizes, heterogeneous age distributions, and inconsistent definitions of severe ENT complications, thereby limiting the robustness and generalizability of the available evidence (1, 11). Potentially relevant predictors—including age, inflammatory biomarkers, imaging findings, and indications for corticosteroid therapy, antibiotic treatment, interventions to secure the airway, or surgery—are inconsistently reported, limiting robust quantitative synthesis and preventing the development of evidence-based risk stratification tools (4, 7).

Previous reviews have primarily summarized the general clinical spectrum, complications, and selected therapeutic aspects of infectious mononucleosis rather than systematically examining life-threatening pediatric ENT manifestations with outcome-specific denominators and explicit certainty assessment (1, 2, 11). Consequently, the frequency and certainty of severe airway and suppurative complications, and the clinical validity of reported warning features, remain poorly defined. The present review therefore asks: (i) which life-threatening ENT complications are documented in pediatric EBV-IM; (ii) which outcome streams can be synthesized quantitatively without combining incompatible populations or denominator directions; (iii) which clinical or laboratory features have been statistically tested versus only descriptively reported; and (iv) how can direct review evidence be distinguished from external clinical/mechanistic context in a cautious, non-validated clinical framework.

Accordingly, this review focuses on two systematically supported ENT domains—clinically significant upper-airway obstruction/airway compromise and suppurative/deep neck complications—while retaining EBV-HLH only as a clinically relevant systemic differential considered in the Discussion.

## 2 Methods

### 2.1 Study design

This systematic review and meta-analysis was reported in accordance with the Preferred Reporting Items for Systematic Reviews and Meta-Analyses (PRISMA 2020) statement (12). Methodological conduct was additionally informed by the Cochrane Handbook for Systematic Reviews of Interventions (13). The review used study-specific sampling frames and denominator directions throughout and restricted quantitative synthesis to clinically compatible populations, denominators, and outcome definitions.

We therefore conducted a systematic review and meta-analysis to characterize severe UAO and suppurative/deep neck complications using study-specific denominators, identify reported candidate warning features, summarize diagnostic and therapeutic approaches, critically appraise methodological quality using design-specific JBI tools (14), assess outcome-level certainty using GRADE (15), and develop a clearly labelled *post-hoc* evidence-informed framework for clinical interpretation.

### 2.2 Review question (PICO)

The review question was formulated according to the Population, Exposure, Comparator, Outcome (PICO) framework (16). The population comprised children and adolescents (0–18 years) with EBV-IM. The exposures/outcomes of interest were severe ENT complications, principally UAO and suppurative complications including peritonsillar, parapharyngeal and retropharyngeal abscesses and other deep neck space infections. Because the available evidence consisted almost exclusively of observational studies, a comparator was not mandatory. EBV-HLH was not included as a systematically searched ENT outcome and was retained only as contextual external evidence relevant to systemic deterioration in EBV infection.

### 2.3 Protocol

A review protocol and eligibility framework were developed before literature screening. The review was not prospectively registered. The complete reproducible PubMed/MEDLINE search strategy, including the final search string, date limits, and eligibility-related search details, is provided in Supplementary Appendix 1.

### 2.4 Eligibility criteria

Eligible studies were English-language original publications reporting acute EBV-associated IM with severe or potentially life-threatening ENT manifestations in children/adolescents or in mixed-age clinical series containing relevant pediatric/adolescent observations, published between January 1, 1990 and September 8, 2026. Eligible designs included prospective or retrospective observational studies and case series comprising at least four patients. Evidence was classified after extraction as direct pediatric evidence when the analyzed cohort was restricted to patients aged ≤18 years, and as mixed-age supportive evidence when pediatric/adolescent observations were embedded within a broader age range and could not be fully separated. Mixed-age studies were retained because they contained clinically relevant ENT outcome data, but their pediatric indirectness was explicitly carried into interpretation and GRADE rather than masked by a retrospective ‘predominantly pediatric’ threshold. For diagnostic eligibility, acute EBV-IM was accepted when the original investigators explicitly diagnosed infectious mononucleosis using EBV-specific serology (for example, VCA-IgM), heterophile-antibody/Monospot testing, or a stated clinical-laboratory case definition compatible with acute IM (for example, a typical clinical syndrome with lymphocytosis or atypical lymphocytes). Studies reporting only past EBV exposure or isolated EBV detection without a clinical diagnosis of acute IM were not eligible. Because diagnostic methods varied across older cohorts, source-specific ascertainment was preserved and reported study by study rather than retrospectively imposing a single laboratory threshold. The ≥4-patient threshold was used to limit reliance on isolated anecdotal reports while retaining evidence on uncommon severe complications. Single case reports, reviews, meta-analyses, editorials, letters without extractable original cohort data, conference abstracts without sufficient data, animal studies, and non-English publications were excluded. Eligibility was determined from the underlying study design and extractable cohort rather than bibliographic publication-type indexing alone.

For evidence categorization, a confirmed life-threatening ENT complication was defined as an ENT manifestation associated with actual or imminent threat to airway patency, respiratory stability, deep neck/septic spread, or another critical ENT-related deterioration requiring or prompting advanced management such as urgent airway intervention, PICU-level monitoring/support, emergency or urgent surgery, or drainage of a deep neck space infection. Severe symptoms or warning presentations (for example dysphagia, tonsillar hypertrophy, persistent fever, neck pain/swelling, or abnormal laboratory findings) were analyzed separately as candidate warning features unless the source study linked them to a confirmed critical event. This operational distinction was used for synthesis and interpretation and was not treated as a validated severity score.

### 2.5 Information sources and search strategy

A systematic literature search was conducted in PubMed/MEDLINE for English-language publications dated January 1, 1990 through September 8, 2026, without geographical restriction. PubMed/MEDLINE was the sole bibliographic database used for the systematic search. The complete reproducible search string is provided in Supplementary Appendix 1. Reference lists of included studies and relevant reviews were checked for potentially eligible reports. No systematic grey-literature search was undertaken; this is acknowledged as a limitation.

To maximize study identification, the reference lists of all eligible articles and relevant review papers were manually screened to identify additional studies meeting the predefined eligibility criteria.

### 2.6 Study selection

Sara Torretta (ST) and Giulia De Lucia (GDL) independently screened titles and abstracts against the predefined eligibility criteria and assessed potentially eligible reports in full text. Disagreements were first resolved through direct discussion between the two reviewers; if consensus was not reached after adequate discussion, the final eligibility decision was made by ST in her role as senior investigator. Eligibility was based on the underlying study design and extractable population rather than publication-type indexing alone. Reasons for full-text exclusion were documented. The study-selection process is reported according to PRISMA 2020 (Figure 1).

**Figure 1:**
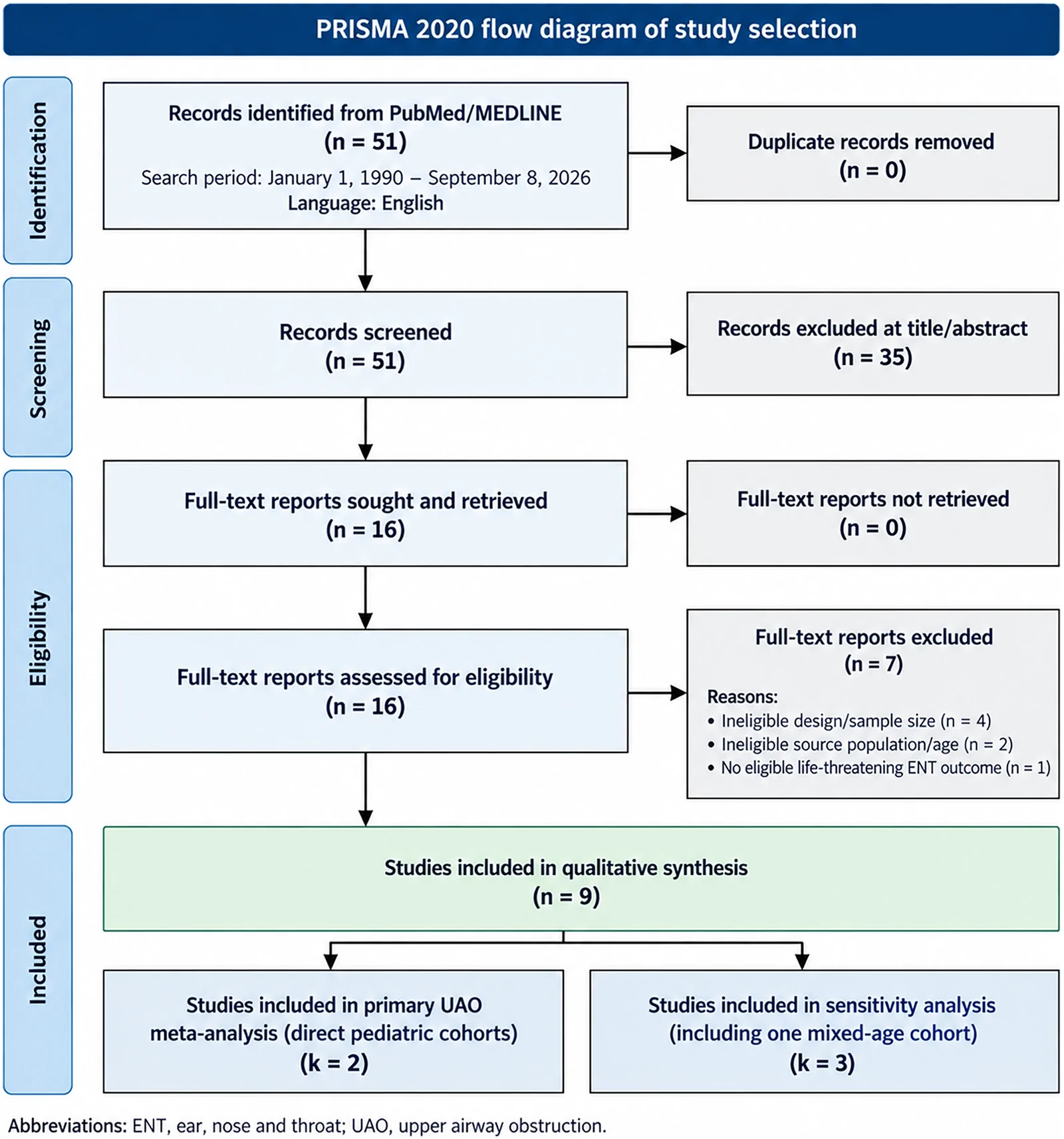
PRISMA 2020 flow diagram of study selection. PubMed/MEDLINE identified 51 records and no duplicates were present. After title/abstract screening, 16 reports underwent full-text assessment and 9 studies were included. Seven full-text reports were excluded: ineligible design or sample size below the prespecified threshold (*n* = 4), ineligible source population or age composition (*n* = 2), and absence of an eligible life-threatening ENT outcome (*n* = 1). Manual citation searching of included studies and relevant reviews identified no additional eligible records (*n* = 0).

### 2.7 Data extraction

Data were extracted independently by two reviewers using a standardized electronic data extraction form developed specifically for this review. All extracted variables were subsequently verified for accuracy and consistency before evidence synthesis.

Extracted variables included study characteristics (first author, publication year, country, study design, and sample size); patient demographics (age, sex, and age range); diagnostic criteria for EBV-IM; clinical presentation; laboratory findings [white blood cell count, atypical lymphocytes, neutrophil count, C-reactive protein (CRP), erythrocyte sedimentation rate, ferritin, aspartate aminotransferase, alanine aminotransferase, lactate dehydrogenase, and platelet count]; imaging findings; medical treatment (corticosteroids, antibiotics, and antiviral therapy); airway interventions; surgical procedures; intensive care unit admission; length of hospital stay; mortality; follow-up; and candidate warning features.

When quantitative synthesis was not feasible because of incomplete or heterogeneous reporting, the available evidence was synthesized narratively.

### 2.8 Definition and categorization of candidate warning features

Because no standardized or validated prediction rule for life-threatening ENT complications of pediatric IM is available, candidate warning features were handled as an exploratory evidence stream rather than as a prespecified predictor set. Features were abstracted when the original studies reported clinical, laboratory, radiological, or endoscopic findings potentially associated with severe ENT deterioration. Features supported only by descriptive reporting were retained as descriptive observations; statistically tested associations were reported separately with their original effect information or *P* values.

During data extraction, candidate warning features were collected using the source authors' terminology. For descriptive synthesis, they were grouped by investigator consensus into clinical presentation, laboratory/microbiological, imaging, and endoscopic domains. This categorization was used only to organize heterogeneous evidence and was not treated as a validated diagnostic or prognostic model.

Outcome data and reported candidate warning features were extracted systematically from eligible studies. During synthesis, warning features were categorized according to whether they were statistically tested, descriptively reported, or derived from external mechanistic/clinical literature; this categorization was interpretive and was not treated as a validated prediction model.

The *post-hoc* evidence-informed framework was developed by Sara Torretta (ST) after completion of data extraction and evidence synthesis, using the prespecified evidence streams together with clearly separated external clinical/mechanistic context. Domain assignment and interpretive wording were discussed with the coauthors; disagreements were addressed through discussion, with final adjudication by ST as senior investigator when consensus was not reached. The framework was not used to determine study eligibility, outcome extraction, or pooling decisions.

### 2.9 Outcome measures

The primary outcomes were life-threatening UAO and suppurative ENT complications, including deep neck space infections and peritonsillar, parapharyngeal, and retropharyngeal abscesses. Secondary outcomes included emergency airway intervention, emergency tonsillectomy, surgical abscess drainage, intensive care admission, bacterial superinfection, reported candidate warning features, medical treatment, length of stay, and mortality. EBV-HLH was not considered a primary or secondary ENT outcome because the final ENT-focused search did not explicitly ascertain HLH literature; any discussion of EBV-HLH was therefore contextual and based on external mechanistic or guideline literature.

### 2.10 Methodological quality assessment

Methodological quality was independently assessed by two reviewers using the JBI critical appraisal tool appropriate to the actual sampling/design of the result interpreted in this review (14, 17). During revision, this appraisal was repeated study by study to avoid applying a uniform checklist across heterogeneous designs. JBI Case Series items were used for disease/condition series without an external analytical comparison, whereas the revised JBI Analytical Cross-Sectional framework was used for contemporaneous analytical comparisons. Responses are reported item-by-item as Yes, No, Unclear, or Not applicable in Supplementary Table S1. No numerical score, percentage of fulfilled items, or universal low/moderate/high cut-off was generated.

### 2.11 Certainty of evidence

The certainty of evidence was independently assessed for each clinically relevant outcome/evidence stream by two reviewers using the Grading of Recommendations Assessment, Development and Evaluation (GRADE) framework (18). Observational evidence started at low certainty. Risk of bias, inconsistency, indirectness, imprecision, and publication/dissemination bias were considered explicitly, without converting study-level JBI responses into a GRADE score. Importantly, certainty that UAO is a clinically documented complication was distinguished from certainty in any single numerical frequency estimate. Outcome-level judgments and domain-specific rationales are reported in Supplementary Table S2.

### 2.12 Statistical analysis

UAO was the only evidence stream for which a narrowly defined quantitative synthesis was judged defensible after denominator-level audit. The primary meta-analysis combined two directly pediatric cohorts of hospitalized patients with IM—Wohl and Isaacson (19) and Balasubramanian et al. (20)—and estimated the proportion with clinically significant UAO/airway compromise. Study proportions were transformed to the logit scale and pooled using a random-effects model with restricted maximum likelihood (REML) estimation of between-study variance. Wald-type 95% confidence intervals were used for the principal pooled estimate. Because only two studies contributed, Hartung-Knapp-Sidik-Jonkman (HKSJ) confidence intervals were examined as a sensitivity analysis. Heterogeneity was described using Cochran's Q, I^2^, and *τ*^2^, with explicit caution regarding their instability with very small k. A sensitivity meta-analysis added the mixed-age Chan and Dawes cohort (5) because its denominator and clinical endpoint were sufficiently similar for robustness assessment; its age-related indirectness was retained in GRADE. No mixed-age cohort contributed to the primary pediatric meta-analysis. Ganzel et al. (4) were excluded from pooling because the relevant denominator was restricted to a severity-enriched severe-pharyngotonsillitis subgroup; Stevenson et al. (3) measured rescue tonsillectomy for persistent severe UAO rather than UAO occurrence; and Gaini et al. (21) comprised purposively selected catastrophic cases. These decisions are summarized in Supplementary Table S3.

Study-level proportions were displayed with Wilson 95% confidence intervals. The pooled UAO estimate and sensitivity analysis were displayed separately from non-poolable study-specific outcomes. No prediction interval was calculated because the primary and sensitivity models contained only two and three studies, respectively.

No meta-regression, funnel-plot analysis, or Egger regression was performed because the UAO meta-analysis contained only two studies in the primary model and three in sensitivity analysis, and no other outcome had enough clinically comparable studies. Publication/dissemination bias was considered qualitatively within GRADE.

Outcomes that were not suitable for quantitative synthesis because of insufficient data or substantial clinical or methodological heterogeneity were synthesized narratively.

Statistical analyses were performed using R (R Foundation for Statistical Computing, Vienna, Austria), primarily employing the meta and metafor packages. A two-sided *P* value <0.05 was considered statistically significant.

Outcome-specific decisions regarding quantitative synthesis are summarized in Supplementary Table S3.

## 3 results

3

### 3.1 Study selection

The final PubMed/MEDLINE search performed on September 8, 2026 identified 51 records. No duplicate records were present in the PubMed export. All 51 records underwent title/abstract screening and 35 were excluded. Sixteen reports were sought and all were retrieved for full-text assessment; seven were excluded because of ineligible study design or sample size below the prespecified threshold (*n* = 4), ineligible source population or age composition (*n* = 2), or absence of an eligible life-threatening ENT outcome (*n* = 1). Nine studies were included in the final evidence synthesis (Figure 1). No aggregate patient total was calculated because the included studies used non-interchangeable sampling frames and denominator directions. Manual citation searching of included studies and relevant reviews identified no additional eligible records (*n* = 0).

The final evidence base comprised nine observational studies published between 1992 and 2026.

### 3.2 Characteristics of included studies

The final evidence base comprised retrospective cohort studies and retrospective case series. Study populations, age composition, clinical setting, and denominator structures differed substantially; therefore, study-specific denominators rather than an aggregate N are used throughout (Table 1).

**Table 1:** Characteristics of included studies.

Study	Design/setting	Population	Age composition	Key extractable outcomes	Main methodological limitation
Stevenson et al., 1992 (3)	Retrospective case series; admitted IM tonsillopharyngitis	25 admitted patients; median 19 y (range 4−33); 15 M/10 F	Mixed pediatric/adult	Steroids 15/25; acute tonsillectomy 6/25; PTA at operation 3/6; intratonsillar abscess in all 6 operated patients.	Mixed-age selected admitted/surgical pathway; retrospective design.
Ganzel et al., 1996 (4)	Retrospective consecutive pediatric IM admissions	109 IM admissions; 60 severe pharyngotonsillitis; mean 9.9 y (range 1–17) in severe subgroup; 33 M/27 F	Pediatric	Severe pharyngotonsillitis 60/109; moderate-to-severe airway compromise 36/60; severe UAO treated with parenteral steroids 29/60; surgery 3/60.	Retrospective design; UAO ascertainment is reported within the severity-enriched 60-patient pharyngotonsillitis subgroup, so 36/109 must not be inferred.
Arkkila et al., 1998 (6)	Retrospective comparative ENT series	928 PA patients; 64 severe pharyngeal IM; 15 IM + PA; IM + PA mean 19 y (15–28)	Adolescent/young adult enriched	IM among PA 15/928; PA among severe pharyngeal IM 15/64; LOS IM + PA 3.1 days; postoperative hemorrhage 1.	ENT referral series; adolescent/young adult enriched.
Chan & Dawes, 2001 (5)	Retrospective chart review plus telephone follow-up	76 IM admissions; 36 IM pharyngotonsillitis; average age 17 y (range 3–27); 20 M/16 F	Mixed pediatric/young adult	UAO 9/36; acute tonsillectomy 7/36; UAO diagnosis not standardized; no anesthetic difficulties or bleeding.	Mixed-age cohort; UAO definition not standardized.
Balasubramanian et al., 2012 (20)	Retrospective pediatric hospital record review	33 hospitalized children; age 9 months−15 y; 22 M/11 F	Pediatric	Complications 10/33; UAO 4/33; HLH 4/33; septic shock 2/33; mortality 0/33; fever >14 days associated with complications.	Brief pediatric hospital report; limited granular reporting.
Danstrup & Klug, 2019 (7)	Retrospective ENT observational study	257 IM with AT/PTA; 220 AT, 37 PTA; median age 17 y (range 2–46); 58% male	Mixed pediatric/adult	PTA 37/257; positive cultures 16/57 AT and 16/32 PTA; neutrophils higher in PTA than AT (*p* = 0.016).	Large retrospective ENT cohort; mixed age and referral selection.
Levi et al., 2020 (8)	Retrospective pediatric CT/tested cohort	100 children with CT and IM testing; 15 PTA; 29 ITA	Pediatric selected by CT/testing	PTA EBV-positive 4/15; ITA EBV-positive 8/29; bilateral ITA EBV-positive 5/12 vs. unilateral 3/17; source reports *p* = 0.218 in abstract and *p* = 0.254 in Results (both non-significant).	Selected by CT and IM testing; unsuitable for IM-complication prevalence.
Gaini et al., 2026 (21)	Published pediatric case series of catastrophic ENT IM	4 selected severe cases among 35 severe IM hospitalizations; age 5–16 y; 2 M/2 F	Pediatric selected catastrophic cases	Respiratory distress cases 1–2; retropharyngeal abscess case 4; intubation cases 1–3; tracheostomy case 3. EBV-HLH death in case 3 retained only as systemic contextual evidence, not an ENT frequency outcome.	Selected catastrophic cases; excellent phenotype detail but high selection bias for prevalence.
Wohl & Isaacson, 1995 (19)	Retrospective pediatric inpatient chart review	45 identified; 36 charts available; median 11 y (range 2–18)	Pediatric	ENT airway evaluation for UAO 11/36; age-stratified descriptive data; airway management including stenting/PICU/urgent T&A	Single-center retrospective design; 36/45 charts available; inpatient selection; non-prospective UAO ascertainment.

Study characteristics are reported as extracted from the original publications. Study names correspond to references (3–8, 19–21). IM, infectious mononucleosis; EBV, Epstein–Barr virus; ENT, ear, nose and throat; UAO, upper airway obstruction; PTA, peritonsillar abscess; ITA, intratonsillar abscess; PICU, pediatric intensive care unit; T&A, tonsillectomy and adenoidectomy.

Substantial variability was observed in study populations and inclusion criteria. The revised evidence base includes fully pediatric cohorts, mixed-age cohorts with pediatric/adolescent relevance, ENT referral populations, abscess cohorts in which EBV positivity was assessed, and highly selected severe presentations. Wohl and Isaacson (1995) (19) contributed an entirely pediatric retrospective inpatient cohort: 45 patients aged ≤18 years with acute IM were identified, of whom 36 charts were available for analysis; median age was 11 years (range, 2–18 years). This heterogeneity was explicitly incorporated into the synthesis as indirectness rather than obscured by an aggregate patient denominator.

Marked heterogeneity was also observed in the study denominators, reflecting differences in study design, patient selection, clinical setting, and outcome definition. Included cohorts ranged from unselected hospitalized patients with IM to children with severe pharyngotonsillitis, otolaryngology referral cohorts, deep neck space/peritonsillar and parapharyngeal abscess cohorts undergoing EBV testing, and highly selected patients presenting with life-threatening complications. Accordingly, quantitative findings are reported as study-specific estimates throughout the following sections. The principal outcome definitions and denominator structures are summarized in Table 2 and underpin the narrative synthesis presented below.

**Table 2:** Extracted outcome-level data with wilson 95% confidence intervals.

Study	Outcome	n/N	Rate (%)	Wilson 95% CI	Denominator and caution
Stevenson et al., 1992 (3)	Steroid-treated severe symptoms	15/25	60.0	40.7–76.6	Mixed-age IM tonsillopharyngitis admissions; treatment indicator.
Stevenson et al., 1992 (3)	Acute tonsillectomy for persistent severe UAO	6/25	24.0	11.5–43.4	Mixed-age; rescue surgery/failure of conservative-steroid pathway.
Stevenson et al., 1992 (3)	Peritonsillar abscess at operation	3/6	50.0	18.8–81.2	Operated-patient denominator only.
Ganzel et al., 1996 (4)	Severe pharyngotonsillitis among IM admissions	60/109	55.0	45.7–64.1	Pediatric hospitalized IM denominator.
Ganzel et al., 1996 (4)	Severe UAO treated with parenteral corticosteroids	29/60	48.3	36.2–60.7	Severe pharyngotonsillitis subgroup.
Ganzel et al., 1996 (4)	Surgical intervention	3/60	5.0	1.7–13.7	Severe pharyngotonsillitis subgroup.
Arkkila et al., 1998 (6)	IM among all PA patients	15/928	1.6	1.0–2.6	Reverse denominator; not IM complication prevalence.
Arkkila et al., 1998 (6)	PA among severe pharyngeal IM in ENT	15/64	23.4	14.7–35.1	ENT-referred severe IM denominator.
Chan & Dawes, 2001 (5)	Upper airway obstruction	9/36	25.0	13.8–41.1	IM pharyngotonsillitis admissions; UAO definition not standardized.
Chan & Dawes, 2001 (5)	Total acute tonsillectomy	7/36	19.4	9.8–35.0	IM pharyngotonsillitis admissions.
Balasubramanian et al., 2012 (20)	Any complication	10/33	30.3	17.4–47.3	Hospitalized pediatric EBV-IM.
Balasubramanian et al., 2012 (20)	Upper airway obstruction	4/33	12.1	4.8–27.3	Hospitalized pediatric EBV-IM.
Danstrup & Klug, 2019 (7)	PTA among IM patients presenting with AT/PTA	37/257	14.4	10.6–19.2	ENT-selected mixed-age cohort.
Danstrup & Klug, 2019 (7)	Positive cultures in AT	16/57	28.1	18.1–40.8	Culture subset only.
Danstrup & Klug, 2019 (7)	Positive cultures in PTA	16/32	50.0	33.6–66.4	Culture subset only.
Levi et al., 2020 (8)	PTA EBV-positive	4/15	26.7	10.9–52.0	PTA denominator, not IM denominator.
Levi et al., 2020 (8)	ITA EBV-positive	8/29	27.6	14.7–45.7	ITA denominator, not IM denominator.
Gaini et al., 2026 (21)	Selected catastrophic cases among severe IM admissions	4/35	11.4	4.5–26.0	Selected cases; not prevalence for all IM.
Gaini et al., 2026 (21)	Respiratory distress among selected cases	2/4	50.0	15.0–85.0	Clinical phenotype only.
Gaini et al., 2026 (21)	Retropharyngeal abscess among selected cases	1/4	25.0	4.6–69.9	Clinical phenotype only.
Wohl & Isaacson, 1995 (19)	ENT airway evaluation for UAO	11/36	30.6	18.0–46.9	Hospitalized pediatric IM cohort; not population prevalence.
Wohl & Isaacson, 1995 (19)	Airway evaluation, age ≤6 y	5/9	55.6	26.7–81.1	Descriptive age subgroup; not a validated predictor.
Wohl & Isaacson, 1995 (19)	Airway evaluation, age >6 y	6/27	22.2	10.6–40.8	Descriptive age subgroup; not a validated predictor.

Wilson confidence intervals are shown for study-level proportions; denominator direction differs across studies. Values are reported only when numerator, denominator, and outcome definition were extractable. Study names correspond to references (3–8, 19–21). IM, infectious mononucleosis; UAO, upper airway obstruction; PTA, peritonsillar abscess; ITA, intratonsillar abscess; EBV, Epstein–Barr virus; CI, confidence interval.

### 3.3 Methodological quality assessment

The included evidence was methodologically heterogeneous and predominantly retrospective. Risk-of-bias concerns varied by study design and included selection/referral bias, incomplete or non-consecutive case ascertainment, non-standardized outcome definitions, incomplete reporting, and limited control of confounding. Accordingly, methodological appraisal is presented at the study- and domain/item-level rather than summarized by an unvalidated numerical JBI score.

Design-specific item-level JBI assessments for all included studies are reported in Supplementary Table S1. These judgments are used to inform interpretation and outcome-level GRADE, without conversion to a common numerical score or global JBI percentage.

### 3.4 Life-threatening ENT complications

Life-threatening UAO was the most consistently reported ENT complication across the included studies, although its frequency varied according to study population, denominator, and outcome definition (Table 2). A narrowly defined quantitative synthesis was feasible for clinically significant UAO/airway compromise among hospitalized patients with IM: the primary direct-pediatric meta-analysis and the mixed-age sensitivity analysis are shown in Figure 2.

**Figure 2:**
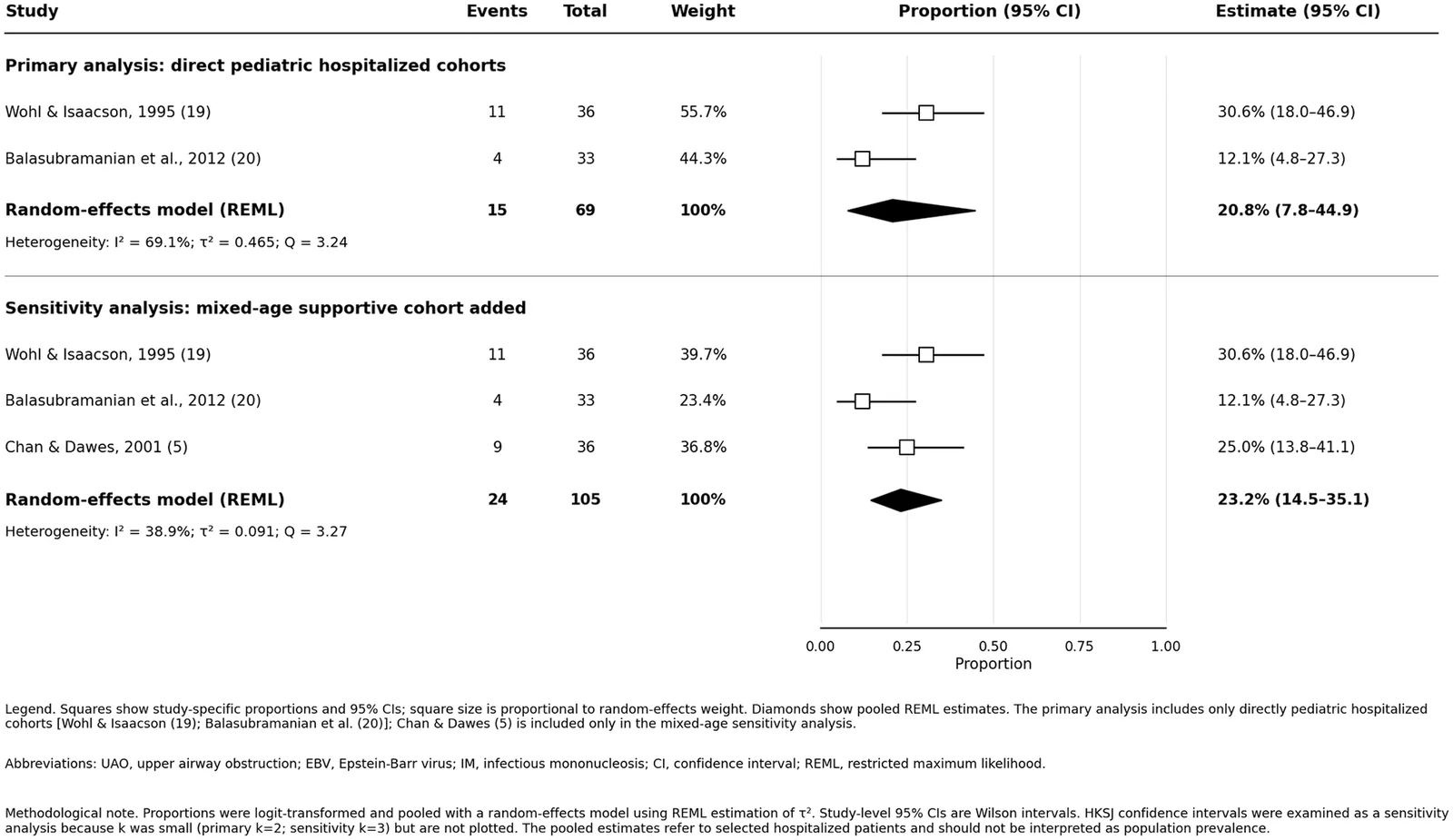
Meta-analysis of clinically significant upper-airway obstruction (UAO)/airway compromise among hospitalized patients with epstein–barr virus infectious mononucleosis. Squares show study-specific proportions with Wilson 95% confidence intervals (CIs), with square size proportional to random-effects weight; diamonds show pooled REML estimates. The primary analysis includes only directly pediatric hospitalized cohorts; Chan and Dawes (2001) is included only in the mixed-age sensitivity analysis. Proportions were logit-transformed and pooled using a random-effects model with REML estimation of tau-squared. Hartung-Knapp-Sidik-Jonkman sensitivity intervals were examined because k was small but are not plotted. Pooled estimates refer to selected hospitalized patients and should not be interpreted as population prevalence. EBV, Epstein–Barr virus; IM, infectious mononucleosis; UAO, upper-airway obstruction; CI, confidence interval; REML, restricted maximum likelihood. Study labels correspond to Wohl and Isaacson (19), Balasubramanian et al. (20), and Chan and Dawes (5).

Among pediatric cohorts, Ganzel et al. (1996) reported that 29 of 60 children with severe IM-associated pharyngotonsillitis (48.3%; Wilson 95% CI, 36.2–60.7) were judged to have severe UAO and received parenteral corticosteroids; surgical intervention was required in three patients (4). Balasubramanian et al. (2012) observed UAO in 4 of 33 hospitalized children with EBV-IM (12.1%; 95% CI, 4.8–27.3) (20). In the fully pediatric inpatient cohort of Wohl and Isaacson (1995), 11 of 36 children (30.6%; Wilson 95% CI, 18.0–46.9) developed noisy breathing, sternal retractions, or nasal flaring of sufficient severity to prompt ENT airway evaluation (19). Airway evaluation was required in 5/9 children aged ≤6 years versus 6/27 older children; this age pattern was descriptive and was not derived from a validated predictive model.

In mixed-age cohorts, Chan and Dawes (2001) reported UAO in 9 of 36 hospitalized patients with IM-associated pharyngotonsillitis (25.0%; 95% CI, 14.0–41.1), although diagnostic criteria for UAO were not standardized (5). Stevenson et al. (1992) reported acute tonsillectomy for persistent severe UAO in 6 of 25 admitted patients with IM-associated tonsillopharyngitis (24.0%; 95% CI, 11.5–43.4) (3).

Primary UAO meta-analysis. The two directly pediatric hospitalized IM cohorts contributed 15 events among 69 children [Wohl and Isaacson (19), 11/36; Balasubramanian et al. (20), 4/33]. The REML logit random-effects pooled proportion of clinically significant UAO/airway compromise was 20.8% (95% CI 7.8–44.9%), with Q = 3.24, I^2^ = 69.1%, and *τ*^2^ = 0.465. Because only two studies contributed, heterogeneity estimates were considered unstable. HKSJ sensitivity analysis produced an extremely wide 95% CI (0.02–99.7%), emphasizing the imprecision of the pooled estimate. When the mixed-age Chan and Dawes cohort (9/36) was added, the sensitivity pooled proportion was 23.2% (95% CI 14.5–35.1%), with Q = 3.27, I^2^ = 38.9%, and *τ*^2^ = 0.091; the corresponding HKSJ 95% CI was 7.1–54.4%. Ganzel, Stevenson, and Gaini remained outside this pooling because their sampling frames or endpoints did not estimate the same UAO occurrence quantity.

Among the four highly selected pediatric patients with life-threatening ENT complications reported by Gaini et al. (2026), respiratory compromise requiring airway assessment occurred in two patients (50.0%; 95% CI, 15.0–85.0) (21).

Deep neck infections were reported using substantially different source populations and denominator definitions (Table 2). Because these estimates did not represent a sufficiently common estimand for pooling, they are retained as study-specific evidence rather than displayed as a main-text quantitative figure.

Studies evaluating patients with IM reported abscess formation as a complication of EBV infection. Arkkila et al. (1998) observed peritonsillar abscess in 15 of 64 patients with severe pharyngeal IM treated in an otolaryngology department (23.4%; 95% CI, 14.8–35.1) (6), whereas Gaini et al. (2026) reported one retropharyngeal abscess among four pediatric cases with life-threatening complications (21).

Danstrup and Klug (2019) evaluated 257 patients with IM who presented with acute tonsillitis (*n* = 220) or peritonsillar abscess (*n* = 37); thus, peritonsillar abscess accounted for 37/257 of this ENT-selected IM cohort (14.4%; Wilson 95% CI, 10.6–19.2), rather than representing the prevalence of IM among abscess patients (7). Levi et al. (2020) used the opposite denominator direction, evaluating a CT- and EBV-tested pediatric cohort and reporting EBV positivity in 4/15 patients with peritonsillar abscess and 8/29 with intratonsillar abscess (8). These estimates were therefore not treated as directly comparable.

Given the substantially different source populations and denominator definitions, these findings are presented separately and were not considered suitable for quantitative pooling.

### 3.5 Candidate clinical warning features

Candidate warning features reported across the included studies are summarized in Supplementary Table S4. These observations should not be interpreted as validated predictors or as a clinical prediction rule because sensitivity, specificity, likelihood ratios, calibration, discrimination, and independent predictive effects were generally unavailable. Statistically tested associations are reported separately in Section 3.6 and Supplementary Table S5.

Frequently reported candidate warning features included progressive dysphagia or poor oral intake, respiratory distress/UAO, marked tonsillar hypertrophy, persistent or prolonged fever, and neck pain or cervical swelling. Laboratory abnormalities such as neutrophilia and elevated CRP were inconsistently reported. Hyperferritinemia and cytopenias relate primarily to systemic EBV-HLH and are therefore not included as systematically supported ENT warning features.

### 3.6 Laboratory and microbiological findings

Laboratory and microbiological findings were heterogeneous across the included studies and were insufficient to support quantitative synthesis.

Prolonged fever represented the only clinical variable significantly associated with complications, with Balasubramanian et al. (2012) reporting an association between fever lasting more than 14 days and adverse outcomes (Fisher's exact test, *P* < 0.05) (20).

Among laboratory parameters, Danstrup and Klug (2019) found significantly higher absolute neutrophil counts in patients with peritonsillar abscess than in those presenting with acute tonsillitis (*P* = 0.016), whereas CRP concentrations did not differ significantly between the two groups (7).

Levi et al. (2020) observed no significant difference in EBV positivity between unilateral and bilateral intratonsillar abscesses; the publication reports *P* = 0.218 in the abstract and *P* = 0.254 in the Results section. Both values are non-significant, and the discrepancy is reported transparently without choosing one value over the other (8).

Case-level data reported by Gaini et al. (2026) described distinct laboratory profiles according to the reported clinical presentation (21). Patients with suppurative complications showed leukocytosis, marked neutrophilia and markedly elevated CRP concentrations, whereas the patient diagnosed with EBV-HLH presented with hyperferritinaemia and cytopenias.

Statistically tested clinical, laboratory, biochemical, and microbiological findings extracted from the included studies are summarized in Supplementary Table S5.

### 3.7 Imaging findings

Imaging findings were reported primarily in studies investigating deep neck infections or life-threatening complications. Levi et al. (2020) included only patients who underwent contrast-enhanced computed tomography (CT) of the head and neck, enabling detailed characterization of peritonsillar and intratonsillar abscesses (8). Gaini et al. (2026) reported contrast-enhanced CT findings in patients with UAO and retropharyngeal abscess and described additional imaging studies in selected patients with systemic complications (21).

Across the included studies, imaging was primarily used to confirm deep neck infection, assess the extent of airway involvement, and support surgical planning. Owing to marked heterogeneity in imaging indications, acquisition protocols, and reported outcomes, quantitative synthesis was not feasible.

### 3.8 Therapeutic management

Therapeutic approaches varied substantially across the included studies and reflected differences in disease severity, institutional practice, and study design (Supplementary Table S6). Treatment indications, regimens, and outcomes were therefore synthesized descriptively and were not presented as a separate main-text figure.

Supportive treatment was reported in several studies. Systemic corticosteroids were commonly administered to patients with severe pharyngotonsillar inflammation or respiratory compromise, although indications and regimens varied. Wohl and Isaacson (1995) additionally described pediatric airway management ranging from observation and temporary nasopharyngeal stenting to intensive-care support and urgent tonsilloadenoidectomy in selected severe cases (19). These data are descriptive and do not establish comparative treatment effectiveness.

Antibiotic therapy was frequently prescribed when bacterial superinfection or deep neck infection was suspected. Danstrup and Klug (2019) reported that bacterial coinfection was not systematically demonstrated among patients with complicated IM despite the frequent use of antibiotics (7). Antiviral therapy was reported only sporadically and inconsistently, precluding any meaningful assessment of its effectiveness or evidence-based recommendation for routine use.

Surgical and advanced airway interventions included acute tonsillectomy, tonsillotomy, abscess tonsillectomy, abscess drainage, endotracheal intubation, and tracheostomy in selected patients with severe airway compromise or advanced deep neck infection.

Because treatment indications, drug regimens, dosages, treatment duration, and surgical criteria varied considerably across studies, quantitative synthesis of therapeutic interventions was not considered appropriate.

### 3.9 Quantitative synthesis

Quantitative synthesis was evaluated separately for each outcome (Supplementary Table S3). After denominator-level audit, UAO was the only evidence stream meeting the threshold for a narrowly defined meta-analysis. The primary pediatric model pooled Wohl and Isaacson (19) with Balasubramanian et al. (20); Chan and Dawes (5) was added only in sensitivity analysis because of mixed-age indirectness. All suppurative/deep neck, biomarker, warning-feature, imaging, and treatment evidence remained non-poolable because of incompatible populations, denominator directions, sampling frames, or outcome definitions. No overall aggregate patient denominator was calculated across the review.

Formal funnel-plot and Egger analyses were not performed because no individual outcome had a sufficient number of clinically comparable studies for meaningful interpretation. Potential publication/dissemination bias was considered qualitatively in the GRADE assessment.

### 3.10 Risk of bias and certainty of evidence

Methodological quality varied across the nine studies. Principal limitations included retrospective design, referral/inpatient selection, mixed pediatric and adult populations in some reports, non-standardized UAO definitions, selected CT-based abscess cohorts, incomplete chart retrieval, and highly selected severe case series. Wohl and Isaacson (19) was considered to have moderate methodological concern because 36 of 45 identified pediatric charts were available, the study was retrospective and single-center, and UAO ascertainment was not prospectively standardized. Detailed domain-level JBI judgments are provided in the Supplementary Materials.

According to GRADE, certainty remained low that UAO represents a clinically important complication in hospitalized pediatric IM. Certainty in the pooled numerical proportion was very low because only two directly pediatric studies contributed to the primary meta-analysis, with substantial imprecision and unstable heterogeneity estimates; the broader three-study sensitivity analysis additionally introduced mixed-age indirectness. Certainty remained very low for deep neck space infection/abscess frequency, candidate clinical warning features, laboratory/microbiological markers, and comparative treatment effectiveness (Supplementary Table S2).

## 4 Discussion

### 4.1 Interpretation of severe ENT manifestations

This systematic review identifies two principal severe ENT evidence domains in pediatric EBV-IM: clinically significant UAO/airway compromise and suppurative/deep neck complications. UAO was the only outcome that supported quantitative synthesis: two directly pediatric hospitalized cohorts yielded a pooled proportion of 20.8% (95% CI 7.8–44.9%; k = 2), with a similar point estimate after addition of one mixed-age cohort in sensitivity analysis (23.2%, 95% CI 14.5–35.1%; k = 3). These hospital-based estimates are imprecise and should not be interpreted as population prevalence. The remaining evidence is best interpreted through study-specific denominators and clinical context rather than pooled frequencies.

The clinical findings can be interpreted alongside established EBV immunobiology, but the included studies did not directly test molecular mechanisms. The framework in Figure 3 therefore organizes the observed ENT evidence and external biological context without proposing a new disease classification.

**Figure 3:**
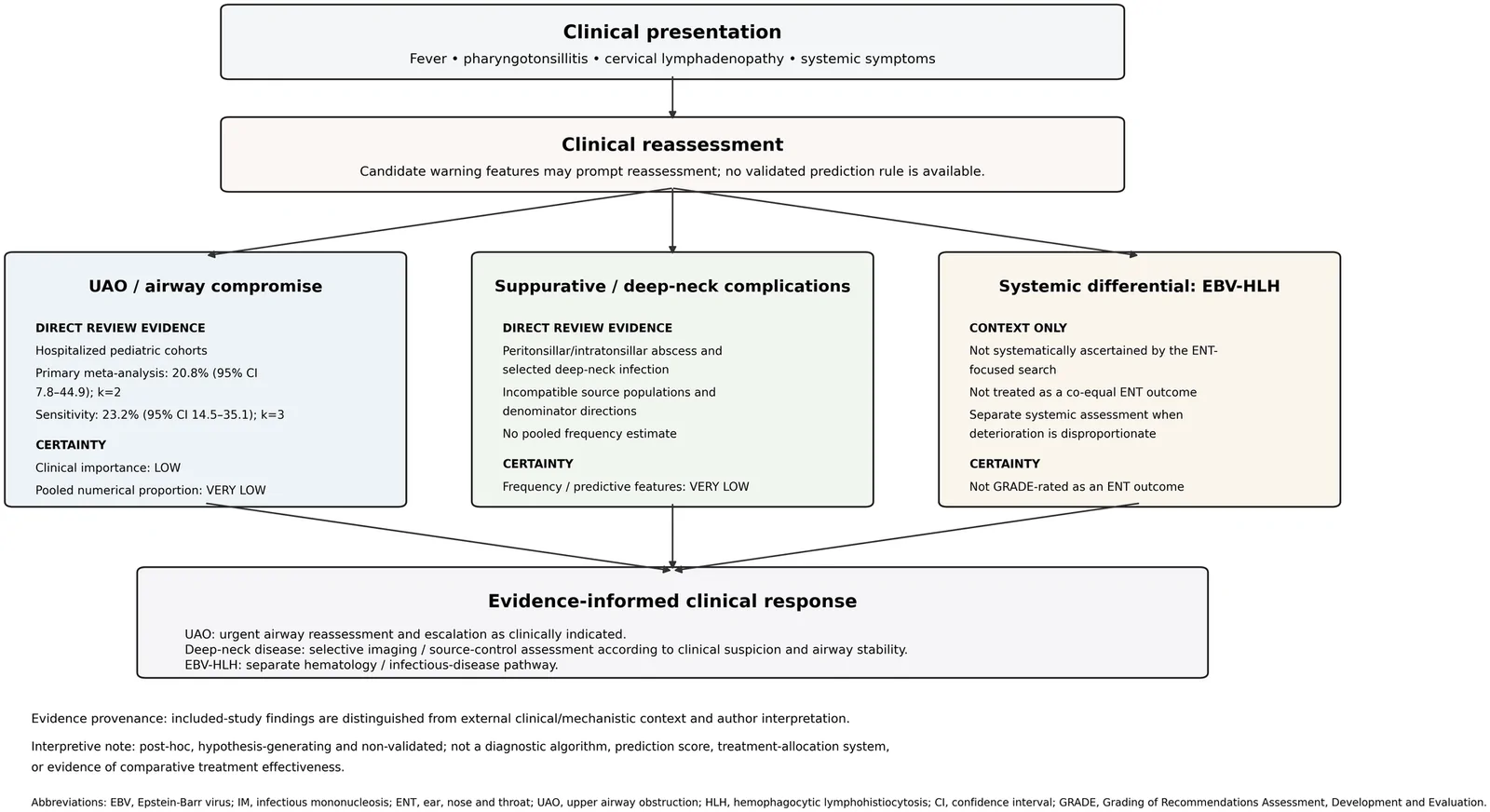
Proposed evidence-informed clinical framework for severe pediatric EBV infectious mononucleosis. The framework separates two ENT evidence domains—UAO and suppurative/deep neck complications—from EBV-HLH, which is shown only as a systemic differential diagnosis. Included-study findings are distinguished from external clinical/mechanistic context and author interpretation. The framework is post-hoc, hypothesis-generating, and non-validated; it is not a diagnostic algorithm, prediction score, treatment-allocation system, or evidence of comparative treatment effectiveness. EBV, Epstein–Barr virus; IM, infectious mononucleosis; ENT, ear, nose and throat; UAO, upper-airway obstruction; HLH, hemophagocytic lymphohistiocytosis; CI, confidence interval; GRADE, Grading of Recommendations Assessment, Development and Evaluation.

#### 4.1.1 From clinical complications to evidence-informed domains

Primary EBV infection produces marked lymphoid activation through interactions between viral persistence and host immune responses (2, 22, 23). These mechanisms provide biological context for severe pharyngeal inflammation, but the included clinical studies did not measure infected-cell lineage, cytokine profiles, viral burden, or tissue immune activation. Mechanistic statements are therefore external contextual evidence rather than findings of the systematic review.

The recurrent clinical patterns are consequently organized as pragmatic evidence domains rather than validated biological phenotypes: a UAO/airway-compromise domain and a suppurative/deep neck domain. This distinction reflects the clinical outcomes and denominator structures actually captured by the included studies.

Figure 3 summarizes this evidence-informed organization and distinguishes direct review findings from external contextual knowledge and clinical interpretation.

#### 4.1.2 UAO domain: clinical interpretation and external biological context

UAO was the most consistently documented severe ENT manifestation. The direct pediatric evidence supports its clinical importance in hospitalized children, although no study established a molecular phenotype or validated a prediction model.

External EBV immunobiology provides a plausible explanation for marked lymphoid enlargement and inflammatory edema within Waldeyer's ring during acute IM (1, 2, 22, 23). Clinically, progressive dysphagia, muffled voice, stridor, increasing respiratory effort, and inability to manage secretions accompany significant airway compromise in the reported series (3–5, 19, 20). These manifestations warrant prompt reassessment, while their diagnostic performance remains unquantified.

Airway assessment takes priority when respiratory compromise is suspected. Cross-sectional imaging should be reserved for a specific clinical question, particularly suspected suppurative/deep neck disease, and must not delay stabilization (24, 25).

#### 4.1.3 Suppurative/deep neck complication domain

Suppurative/deep neck complications constitute a second clinically important evidence stream. Peritonsillar, intratonsillar, and selected deep neck abscesses were reported, but source populations and denominator directions differed substantially, precluding a common pooled frequency.

Danstrup and Klug (7) reported higher absolute neutrophil counts in peritonsillar abscess than in acute tonsillitis, whereas Levi et al. (8) found no significant association between EBV positivity and unilateral versus bilateral intratonsillar abscess; Gaini et al. (21) provided selected case-level microbiological and inflammatory data. No single pathogen or biomarker was reproducibly associated with complicated EBV-IM.

Persistent or recurrent fever with focal symptoms such as unilateral odynophagia, trismus, cervical swelling, uvular deviation, or worsening neck pain should prompt targeted evaluation for suppurative disease. Contrast-enhanced CT or MRI may be used selectively according to the clinical question, local availability, and airway stability rather than routinely (24, 25).

#### 4.1.4 EBV-HLH as a clinically relevant systemic differential

EBV-HLH is a systemic hyperinflammatory syndrome rather than an ENT complication and was not systematically ascertained by the ENT-focused search. Its inclusion here is clinically motivated because children initially managed for severe EBV-IM may present with prominent pharyngotonsillar/airway disease and subsequently develop systemic deterioration that requires recognition outside the ENT pathway.

Persistent fever with progressive cytopenias, hyperferritinemia, hepatosplenomegaly, liver dysfunction, coagulopathy, hypofibrinogenemia, hypertriglyceridemia, or disproportionate clinical deterioration should prompt consideration of HLH and multidisciplinary assessment according to established diagnostic criteria (9, 10, 26). EBV-HLH may involve B-, T-, or NK-cell compartments, and hemophagocytosis is neither required nor specific for diagnosis (9, 26).

Accordingly, suspected EBV-HLH requires a separate hematology/infectious-disease and critical-care pathway. The review does not estimate its frequency, prognosis, or treatment effects; the purpose of retaining it in the clinical discussion is to ensure that clinicians managing severe EBV-IM recognize this potentially life-threatening systemic differential.

#### 4.1.5 Evidence-informed synthesis

Taken together, the evidence supports a practical framework centered on the two ENT domains captured by the review, with clinical interpretation informed—but not determined—by external mechanistic literature. Table 3 makes the provenance of each component explicit, and Figure 3 provides the corresponding clinical synthesis.

**Table 3:** Evidence provenance and clinical interpretation of severe EBV-IM manifestations.

Domain	Evidence directly supported by included studies	External/contextual evidence	Clinical interpretation	Certainty/limitation
UAO	Hospitalized pediatric and mixed-age IM series document airway compromise, ENT airway evaluation, corticosteroid-treated severe UAO, intubation/PICU care and selected urgent tonsil surgery. Study-specific denominators remain heterogeneous. (3–5, 19–21).	General pediatric airway assessment principles and contemporary clinical practice inform escalation, endoscopic assessment when feasible, and airway stabilization. (1, 11, 27).	Progressive airway symptoms require urgent reassessment. Flexible nasopharyngolaryngoscopy may be useful when clinically appropriate and safe. Imaging must not delay airway stabilization.	Low certainty that UAO is a clinically important complication in hospitalized pediatric IM; very low certainty for the pooled numerical proportion because k = 2, imprecision, and unstable heterogeneity estimates. No validated prediction rule.
Suppurative/deep neck complications	Peritonsillar abscess, intratonsillar abscess and one selected retropharyngeal abscess are reported, but studies use different sampling frames and denominator directions. Danstrup reports higher neutrophils in peritonsillar abscess vs. acute tonsillitis (*p* = 0.016). (6–8, 21).	Pediatric deep neck infection literature informs selective cross-sectional imaging, recognition of abscess extension, antimicrobial treatment and source control. MRI avoids ionizing radiation but may be less immediately available and can require sedation/anesthesia. (24, 25).	Persistent or focal worsening symptoms (e.g., unilateral odynophagia, trismus, neck swelling/pain, uvular deviation) should prompt evaluation for suppurative disease. Contrast-enhanced CT or MRI should be used selectively according to the clinical question, local availability and airway stability.	Very low certainty for frequency or predictive features. No pooled prevalence; reverse denominators and CT/testing selection preclude a common estimand.
EBV-HLH — systemic context only	Not systematically ascertained by the frozen ENT-focused search and therefore not graded as an ENT outcome. Systemic cases reported within included papers are retained contextually but not used for ENT frequency inference.	HLH diagnostic biology and treatment derive from dedicated hematology/HLH literature. EBV may involve B-, T- or NK-cell compartments; hemophagocytosis is neither required nor specific for diagnosis. (9, 10, 26).	Persistent fever with progressive cytopenias, hyperferritinemia, liver dysfunction, coagulopathy, hepatosplenomegaly or disproportionate systemic deterioration warrants separate HLH evaluation and specialist management.	No ENT GRADE rating. This review cannot estimate EBV-HLH frequency, prognosis or treatment effects.

This table distinguishes findings supported by the included studies from external clinical/mechanistic context and author interpretation. Included-study evidence is supported by references 3–8 and 19–21 as applicable; external/contextual statements are supported by references (1, 2, 9–11, 21–23, 26) as applicable. EBV, Epstein–Barr virus; IM, infectious mononucleosis; UAO, upper airway obstruction; HLH, hemophagocytic lymphohistiocytosis; GRADE, Grading of Recommendations Assessment, Development and Evaluation.

The framework is intended to organize heterogeneous evidence and guide clinical attention to airway compromise and suppurative disease; it is not a validated prediction or treatment-allocation model. Prospective pediatric validation is required before formal risk stratification can be proposed.

### 4.2 Evidence-informed clinical implications

The two ENT evidence domains translate into a focused assessment strategy: progressive airway symptoms require prioritization of airway evaluation, whereas focal or progressive features suggesting deep neck infection warrant targeted investigation for suppurative disease. These pathways combine review evidence with established clinical practice and external guidance.

#### 4.2.1 Candidate clinical warning features

Early recognition of candidate warning features may support timely reassessment, but the included studies did not validate a prediction rule. Progressive dysphagia, muffled voice, inability to manage secretions, stridor, or increasing work of breathing warrant urgent airway reassessment (3–5). Persistent or recrudescent fever with unilateral odynophagia, trismus, cervical swelling, uvular deviation, or worsening neck pain raises concern for suppurative disease (6, 7, 24). No individual finding is diagnostic in isolation.

Systemic features such as persistent fever accompanied by progressive cytopenias, marked hyperferritinemia, hepatosplenomegaly, coagulopathy, hypofibrinogenemia, hypertriglyceridemia, or disproportionate deterioration should instead trigger consideration of HLH according to dedicated external guidance (9, 10, 26).

Table 3 summarizes evidence provenance and clinical interpretation; Figure 3 presents the integrated clinical framework.

#### 4.2.2 Selective diagnostic assessment

Initial assessment should confirm or appropriately investigate acute EBV infection and include a systematic general, oral/oropharyngeal, and ENT examination. Oral and oropharyngeal assessment should include tonsillar enlargement and exudate, palatal petechiae, pharyngeal erythema, gingival inflammation or swelling, oral mucosal ulceration, palatal or uvular asymmetry, swallowing and secretion handling, voice changes, hydration, and respiratory effort (1, 11, 27). These findings should be interpreted as components of a comprehensive examination rather than as a validated prediction rule. Subsequent investigations should be driven by the suspected complication and clinical trajectory rather than applied routinely to every child with IM.

Patients with progressive UAO require repeated clinical airway assessment, with flexible nasopharyngolaryngoscopy when clinically appropriate and feasible. Cross-sectional imaging should not be routine. When deep neck infection is suspected, contrast-enhanced CT or MRI may be selected according to the diagnostic question, local availability, need for speed, likelihood of sedation/anesthesia, and airway stability; CT entails ionizing radiation, whereas MRI avoids radiation and can accurately characterize pediatric deep neck infection but may be less immediately available (24, 25). Imaging must not delay airway stabilization.

#### 4.2.3 Multidisciplinary management

Severe EBV-IM may require multidisciplinary care according to the dominant clinical problem.

Airway instability requires coordination among pediatrics, ENT, anesthesiology, and pediatric critical care (3, 4, 20). Suspected suppurative/deep neck infection may additionally require radiology, microbiology, antimicrobial therapy, and surgical source control when indicated (6, 7, 24, 25). Suspected EBV-HLH requires prompt hematology/infectious-disease and critical-care assessment according to dedicated HLH guidance (9, 10, 26).

#### 4.2.4 Implications and validation needs

The proposed framework is an organizational model for a sparse and heterogeneous evidence base. Its value lies in separating clinically distinct evidence streams and making the provenance of review-derived versus external contextual information explicit.

Prospective multicenter pediatric studies are required to determine whether candidate warning features or combinations of findings have reproducible diagnostic or prognostic value.

### 4.3 Strengths, limitations and future directions

Strengths of this review include explicit separation of evidence streams by source population, denominator direction, and pediatric directness; preservation of clinically relevant non-poolable data; design-specific JBI appraisal; outcome-level GRADE; and restriction of meta-analysis to a clinically compatible UAO estimand.

Important limitations remain. All included studies were observational and predominantly retrospective, with small or selected samples, referral/inpatient selection, and non-standardized definitions of EBV infection, UAO, and suppurative complications. Some cohorts included adults, creating pediatric indirectness; however, no mixed-age cohort contributed to the primary pediatric UAO meta-analysis. Sampling frames and denominator directions were incompatible across several studies, preventing aggregate prevalence estimates. Candidate warning features lacked diagnostic-performance or multivariable validation. The search was restricted to English-language PubMed/MEDLINE records and did not include a systematic grey-literature search; the review was not prospectively registered. The primary meta-analysis contained only two directly pediatric cohorts, making the summary estimate and heterogeneity parameters imprecise, while the three-study sensitivity analysis introduced mixed-age indirectness. Management suggestions should therefore be interpreted as evidence-informed clinical considerations rather than comparative effects demonstrated by this review.

Future research should prioritize prospective multicenter pediatric cohorts using standardized definitions of UAO and suppurative/deep neck complications, harmonized denominator reporting, and prespecified assessment of candidate clinical and laboratory features. Dedicated EBV-HLH evidence should be evaluated separately using an appropriately targeted systematic search.

## 5 Conclusions

Life-threatening ENT complications of pediatric EBV-IM are uncommon in the literature but clinically consequential. UAO is the best-supported severe ENT manifestation: certainty that UAO is a clinically important complication in hospitalized pediatric IM is low, while certainty in the pooled numerical proportion of 20.8% (95% CI 7.8–44.9%) is very low because only two directly pediatric cohorts contributed and imprecision remains substantial. Evidence for suppurative/deep neck complications, candidate warning features, laboratory/microbiological markers, and comparative treatment effects is also very low or quantitatively non-poolable. By distinguishing direct pediatric evidence from mixed-age supportive data and separating review-derived findings from external clinical context, this review provides a structured synthesis to support clinical awareness and hypothesis generation rather than a validated early-recognition or prediction framework.

Prospective multicenter pediatric studies using standardized outcome definitions, consistent denominators, and validated predictive methods are now needed to refine risk assessment and determine which candidate warning features have reproducible clinical utility.

## Data Availability

The original contributions presented in the study are included in the article/Supplementary Material, further inquiries can be directed to the corresponding author.
