# Supplementary material for: Early recognition of life-threatening otolaryngologic complications in pediatric epstein-barr virus infectious mononucleosis: a systematic review and meta-analysis
Source: Front Pediatr. 2026 Oct 8;14:1980308. doi: 10.3389/fped.2026.1980308 (PMC13687337; doi:10.3389/fped.2026.1980308)
Supplement: Supplementary file 1 [file Supplementaryfile1.docx]

Supplementary Materials

Supplementary Appendix 1. Complete Electronic Search Strategy

Database: PubMed/MEDLINE

Search period: January 1, 1990 to September 8, 2026

Language restriction: English

Species restriction: None applied as a separate search filter

Geographical restrictions: None

Final search performed on September 8, 2026. The search retrieved 51 records. No publication-type exclusion filter was applied; eligibility was determined at the study level during screening.

Final frozen PubMed search string

(
"Infectious Mononucleosis"[Mesh]
OR "infectious mononucleosis"[tiab]
OR (mononucleosis[tiab] AND EBV[tiab])
OR ("Epstein-Barr virus"[tiab] AND mononucleosis[tiab])
)
AND
(
child*[tiab]
OR pediatric*[tiab]
OR paediatric*[tiab]
OR adolescent*[tiab]
OR infant*[tiab]
OR "Child"[Mesh]
OR "Adolescent"[Mesh]
)
AND
(
"upper airway obstruction"[tiab]
OR "airway compromise"[tiab]
OR intubation[tiab]
OR tracheotomy[tiab]
OR tracheostomy[tiab]
OR "emergency tonsillectomy"[tiab]
OR "acute tonsillectomy"[tiab]
OR "hot tonsillectomy"[tiab]
OR "peritonsillar abscess"[tiab]
OR "retropharyngeal abscess"[tiab]
OR "parapharyngeal abscess"[tiab]
OR "deep neck infection"[tiab]
)
AND english[Language]
AND ("1990/01/01"[Date - Publication] : "2026/09/08"[Date - Publication])

Study-level eligibility note. Records were assessed according to the prespecified study-level eligibility criteria irrespective of PubMed publication-type indexing. Wohl and Isaacson (19) met eligibility as a retrospective pediatric cohort despite the record's publication-type indexing.

Diagnostic ascertainment note. Acute EBV-IM eligibility accepted source-defined acute infectious mononucleosis based on EBV-specific serology, heterophile-antibody/Monospot testing, or an explicitly stated clinical-laboratory case definition compatible with acute IM. Diagnostic ascertainment varied across studies and was preserved as reported rather than standardized retrospectively to a single laboratory threshold.

Supplementary Table S1. Design-Specific JBI Critical Appraisal. Studies correspond to references 3–8 and 19–21 in the main manuscript.

Item-level appraisal was repeated during the 2026 revision audit using the JBI tool appropriate to the actual sampling/design of the result interpreted in this review. Responses are Yes (Y), No (N), Unclear (U), or Not applicable (NA). No numerical score, percentage of fulfilled items, or universal low/moderate/high threshold was generated. A No or Unclear response identifies a limitation relevant to interpretation and is not a study-exclusion rule.

Part A. JBI Case Series checklist (10 items)

Q1 clear inclusion criteria; Q2 condition measured in a standard/reliable way; Q3 valid identification method; Q4 consecutive inclusion; Q5 complete inclusion; Q6 demographics reported; Q7 clinical information reported; Q8 outcomes/follow-up reported; Q9 site/clinic information reported; Q10 appropriate statistical analysis.

| Study | Q1 | Q2 | Q3 | Q4 | Q5 | Q6 | Q7 | Q8 | Q9 | Q10 |
| --- | --- | --- | --- | --- | --- | --- | --- | --- | --- | --- |
| Stevenson et al., 1992 (3) | Y | U | U | U | U | Y | Y | Y | Y | NA |
| Wohl & Isaacson, 1995 (19) | Y | U | U | Y | N | Y | Y | Y | Y | NA |
| Ganzel et al., 1996 (4) | Y | Y | Y | Y | Y | Y | Y | Y | Y | Y |
| Chan & Dawes, 2001 (5) | Y | Y | Y | Y | Y | Y | Y | Y | Y | NA |
| Balasubramanian et al., 2012 (20) | Y | Y | Y | U | U | Y | Y | Y | Y | Y |
| Gaini et al., 2026 (21) | U | Y | Y | N | N | Y | Y | Y | Y | NA |

Audit notes: Stevenson (3)—mixed-age admitted IM tonsillopharyngitis; ascertainment/completeness not fully explicit. Wohl and Isaacson (19)—all 45 pediatric IM discharges identified but only 36 charts available; serologic confirmation in 32/36. Ganzel et al. (4)—consecutive pediatric admissions with explicit clinical/Monospot/lymphocytosis ascertainment. Chan and Dawes (5)—consecutive IM admissions with mixed pediatric/young-adult age range. Balasubramanian et al. (20)—pediatric hospital record review with limited granular reporting. Gaini et al. (21)—purposively selected catastrophic pediatric cases, appropriate for descriptive phenotype detail but highly selected for frequency inference.

Part B. Revised JBI Analytical Cross-Sectional checklist (8 items)

Q1 inclusion criteria clearly defined; Q2 objective standard criteria for measurement of the condition; Q3 exposure measured validly/reliably; Q4 outcomes measured validly/reliably; Q5 confounders identified; Q6 strategies to deal with confounding stated; Q7 appropriate statistical analysis used; Q8 study subjects and setting described in detail. Responses are Yes (Y), No (N), Unclear (U), or Not applicable (NA).

| Study | Q1 | Q2 | Q3 | Q4 | Q5 | Q6 | Q7 | Q8 |
| --- | --- | --- | --- | --- | --- | --- | --- | --- |
| Arkkila et al., 1998 (6) | Y | Y | Y | Y | Y | Y | U | Y |
| Danstrup & Klug, 2019 (7) | Y | U | U | Y | U | N | Y | Y |
| Levi et al., 2020 (8) | Y | Y | Y | Y | N | N | Y | Y |

Audit notes: Arkkila et al. (6)—two distinct ENT sampling frames; peritonsillar abscess surgically confirmed; estimands 15/928 and 15/64 remain separate. Danstrup and Klug (7)—consecutive admitted IM plus acute tonsillitis/peritonsillar abscess cohort; IM based on lymphocytosis and/or Monospot; no multivariable confounding control. Levi et al. (8)—pediatric CT-and-IM-testing-selected sample; appropriate for within-cohort abscess/EBV comparisons but not for IM-complication prevalence.

Supplementary Table S2. GRADE Outcome-Level Evidence Profile. Study names correspond to references 3–8 and 19–21 in the main manuscript.

Observational evidence started at low certainty. Risk of bias, inconsistency, indirectness, imprecision, and publication/dissemination bias were considered explicitly. No upgrading was applied. Formal funnel-plot/Egger methods were not used because no outcome had enough clinically comparable studies for meaningful interpretation; publication/dissemination bias was considered qualitatively and was not downgraded automatically solely on study count.

| Outcome / evidence stream | Evidence base | Domain-specific GRADE judgments | Final certainty | Interpretation |
| --- | --- | --- | --- | --- |
| UAO as a documented severe ENT complication in hospitalized pediatric IM | Direct pediatric: Ganzel 1996 (4); Wohl 1995 (19); Balasubramanian 2012 (20). Mixed-age supportive: Chan 2001 (5); Stevenson 1992 (3). Gaini 2026 (21) = selected severe-case context only. | Risk of bias: serious concerns, not uniform. Inconsistency: no downgrade for existence of the clinical phenomenon, although severity definitions differ. Indirectness: serious for general-population inference, reduced by three direct pediatric studies. Imprecision: serious. Publication/dissemination bias: uncertain; no additional automatic downgrade. | Low | UAO is a real clinically important complication. This does not establish a single prevalence estimate or validated prediction rule. |
| Pooled numerical proportion of clinically significant UAO / airway compromise | Primary: Wohl 1995 (19) + Balasubramanian 2012 (20) (direct pediatric); sensitivity: + Chan 2001 (5) (mixed-age supportive). | Risk of bias: serious. Inconsistency: serious in the primary model (I²=69.1%), lower in sensitivity (I²=38.9%) but k is very small. Indirectness: serious for general-population inference and increased by mixed-age sensitivity analysis. Imprecision: very serious, particularly with k=2; HKSJ interval extremely wide. Publication/dissemination bias: uncertain and not statistically testable. | Very low | Primary REML pooled proportion 20.8% (95% CI 7.8–44.9%; k=2); sensitivity 23.2% (95% CI 14.5–35.1%; k=3). Interpret as clinically significant airway compromise in selected hospitalized patients, not population prevalence. |
| Deep neck space infection / abscess associated with IM | Arkkila 1998 (6); Danstrup 2019 (7); Levi 2020 (8); Gaini 2026 (21) retropharyngeal abscess as selected severe-case context. | Risk of bias: serious. Inconsistency: serious because studies answer different questions. Indirectness: very serious (reverse denominators, mixed-age ENT populations, selected abscess/CT cohorts). Imprecision: serious. Publication/dissemination bias: uncertain and unusual-presentation reporting is plausible. | Very low | Association is supported in selected settings, but not a pooled pediatric complication rate. |
| Candidate clinical warning features | Descriptive features across studies; statistically tested signals mainly prolonged fever in Balasubramanian and selected laboratory comparisons in Danstrup. | Risk of bias: serious. Inconsistency: serious. Indirectness: serious. Imprecision: very serious; no sensitivity/specificity, likelihood ratios, calibration, discrimination, or external validation. Publication/dissemination bias: uncertain. | Very low | Use candidate warning features/warning features, not validated predictors, score, or clinical prediction rule. |
| Laboratory / microbiological markers | Balasubramanian 2012 (20); Danstrup 2019 (7); Levi 2020 (8); selected case-level Gaini 2026 (21) data. | Risk of bias: serious (selective testing/confounding/exploratory comparisons). Inconsistency: serious. Indirectness: serious. Imprecision: very serious; no common threshold or accuracy estimate. Publication/dissemination bias: uncertain. | Very low | Retain exact reported associations; do not infer pooled biomarker thresholds or diagnostic/prognostic accuracy. |
| Comparative treatment effectiveness | Management experience reported across the included studies; no eligible randomized comparative treatment body for this review question. | Risk of bias: very serious because of confounding by indication and clinician-selected treatment. Inconsistency: serious. Indirectness: serious. Imprecision: serious. Publication/dissemination bias: uncertain; treatment successes in case series may be preferentially reported. | Very low | Management data are descriptive; no comparative efficacy claim. External guidelines/practice recommendations must be cited separately. |

Supplementary Table S3. Quantitative Synthesis Decisions. UAO analyses use Wohl and Isaacson (19), Balasubramanian et al. (20), and Chan and Dawes (5), as specified below.

| Outcome/analysis | Decision | Rationale |
| --- | --- | --- |
| Primary UAO meta-analysis | Performed: k=2 direct pediatric cohorts | REML logit random effects: 20.8% (95% CI 7.8–44.9%); I²=69.1%; τ²=0.465. HKSJ sensitivity interval extremely wide. |
| UAO sensitivity analysis | Performed: + mixed-age Chan & Dawes (5) | REML logit random effects: 23.2% (95% CI 14.5–35.1%); I²=38.9%; τ²=0.091. Mixed-age indirectness retained. |
| Deep neck space infection pooled prevalence | Not performed | Denominator directions are not interchangeable: severe IM ENT referral cohort, IM + acute tonsillitis/peritonsillar abscess cohort, abscess/CT cohort with EBV testing, and selected severe cases. |
| Laboratory warning-feature meta-analysis | Not performed | Markers, thresholds and clinical contexts were not sufficiently harmonizable; tested associations are retained individually. |
| Publication-bias statistical testing | Not performed | No outcome had a sufficient number of clinically comparable studies for meaningful funnel-plot or Egger interpretation. Publication/dissemination bias is considered qualitatively in GRADE. |

After denominator-level reappraisal, one narrowly defined UAO meta-analysis was considered feasible. The primary analysis combined Wohl and Isaacson (19; 11/36) and Balasubramanian et al. (20; 4/33), both directly pediatric hospitalized IM cohorts, using logit-transformed proportions and REML random effects. The pooled proportion was 20.8% (95% CI 7.8–44.9%; Q=3.24; I²=69.1%; τ²=0.465; k=2). HKSJ sensitivity produced a very wide 95% CI (0.02–99.7%). Adding Chan and Dawes (5; 9/36) in a mixed-age sensitivity analysis yielded 23.2% (95% CI 14.5–35.1%; Q=3.27; I²=38.9%; τ²=0.091; k=3; HKSJ 95% CI 7.1–54.4%). Other evidence streams remained non-poolable.

Methodological provenance. The evidence-preservation matrix, design-specific JBI reappraisal and GRADE evidence profile were generated during the 2026 revision audit in response to peer-review concerns. These revision-stage appraisals do not imply that a later JBI tool version had been used before its publication. Raw numerator/denominator data remain preserved independently of appraisal judgments.

**Supplementary Table S4. Candidate Clinical Warning Features Reported Across Included Studies**

Legend. Candidate warning features are descriptive and are not validated predictors or a clinical prediction rule. Abbreviations: UAO, upper airway obstruction; CRP, C-reactive protein; CT, computed tomography. Statistical note: descriptive synthesis only. Reference numbers in parentheses correspond to the main-manuscript reference list.

| **Candidate warning feature** | **Studies reporting** | **Evidence strength** | **Clinical implication** |
| --- | --- | --- | --- |
| Progressive dysphagia / poor oral intake | Stevenson (3); Ganzel (4); Gaini (21) [n=3 studies] | Repeatedly reported; definitions not uniform. | Treat as a bedside warning sign prompting reassessment of hydration, swallowing safety and airway trajectory. |
| Respiratory distress / UAO | Stevenson (3); Ganzel (4); Chan (5); Balasubramanian (20); Gaini (21); Wohl & Isaacson (19) [n=6 studies] | Frequently reported severe ENT signal; definitions and sampling frames heterogeneous. | Urgent ENT/anesthesia evaluation and dynamic monitoring are warranted. |
| Stridor / stertor / apnea / desaturation | Stevenson (3); Chan & Dawes (5) [n=2 studies] | Highly clinically relevant but inconsistently reported. | Escalate immediately as possible impending airway failure. |
| Marked or kissing tonsillar hypertrophy | Ganzel (4); Gaini (21) [n=2 studies] | Important bedside sign; grading not standardized. | Combine with symptoms and oxygenation rather than using tonsil size alone. |
| Persistent/prolonged fever | Balasubramanian (20); Gaini (21) [n=2 studies] | Fever >14 days was associated with complications in one small pediatric series; otherwise descriptive. | Prompt reassessment for complicated IM; systemic EBV-HLH evaluation is a contextual differential when persistent fever is accompanied by cytopenias/hyperferritinemia or systemic deterioration. |
| Neutrophilia / high CRP | Danstrup & Klug (7); Gaini (21) [n=2 studies] | Danstrup reported higher absolute neutrophil count in PTA vs acute tonsillitis (p=0.016); CRP findings were less discriminative. | Supportive, not diagnostic, evidence for bacterial/suppurative assessment. |
| Neck pain/swelling/limited movement | Balasubramanian (20); Arkkila (6); Gaini (21) [n=3 studies] | Deep-neck infection warning sign. | Consider deep neck space infection; imaging should be selective and clinically justified rather than routine. |

**Supplementary Table S5. Statistically Tested Clinical, Laboratory, Biochemical, and Microbiological Findings Reported in the Included Studies**

Legend. Statistically tested findings are reported whether significant or non-significant when relevant to the review question. Only P values explicitly reported in the original studies or verified during extraction are presented. Abbreviations: EBV, Epstein-Barr virus; IM, infectious mononucleosis; ITA, intratonsillar abscess; PTA, peritonsillar abscess. Statistical note: no additional statistical analyses were performed. Reference numbers in parentheses correspond to the main-manuscript reference list.

| **Study** | **Variable/outcome** | **Comparison** | **P value** | **Interpretation** |
| --- | --- | --- | --- | --- |
| Danstrup & Klug, 2019 (7) | Absolute neutrophil count | PTA vs acute tonsillitis | p=0.016 | Higher neutrophil count supported the bacterial/suppurative pathway. |
| Levi et al., 2020 (8) | EBV positivity | Bilateral vs unilateral ITA | p=0.254 in Results; p=0.218 in abstract | No statistically significant association; internal source discrepancy reported transparently. |
| Balasubramanian et al., 2012 (20) | Fever duration >14 days | Complicated vs uncomplicated hospitalized EBV-IM | Fisher exact p<0.05 | Prolonged fever was associated with complications in one pediatric series. |

**Supplementary Table S6. Therapeutic Strategies Reported Across Included Studies**

Legend. Treatment information is descriptive and does not establish comparative effectiveness. Reference numbers in parentheses correspond to the main-manuscript reference list.

| **Treatment domain** | **Studies reporting** | **Extracted interpretation** |
| --- | --- | --- |
| Supportive care | Stevenson (3); Ganzel (4); Chan (5); Balasubramanian (20); Gaini (21); Wohl (19) | Hydration, analgesia/antipyretics and monitoring; details variable. |
| Corticosteroids | Stevenson (3); Ganzel (4); Chan (5); Balasubramanian (20); Gaini (21); Wohl (19) | Used for severe symptoms or airway compromise; dose and indication heterogeneous. |
| Antibiotics | Stevenson (3); Ganzel (4); Arkkila (6); Danstrup (7); Gaini (21) | Frequently used when bacterial complications were suspected; benefit uncertain without confirmed superinfection. |
| Airway intervention | Stevenson (3); Chan (5); Gaini (21) | Intubation, tracheostomy and/or acute surgery in selected severe cases. |
| Surgery | Stevenson (3); Ganzel (4); Arkkila (6); Chan (5); Gaini (21) | Acute tonsillectomy, tonsillotomy or drainage; indications heterogeneous. |

Supplementary presentation note. Tables S4-S6 preserve descriptive warning-feature, statistical-finding, and treatment information removed from the main manuscript to reduce visual redundancy. No study-level value or denominator was changed during this editorial consolidation.
